# Plasma concentrations of leptin at mid-pregnancy are associated with gestational weight gain among pregnant women in Tanzania: a prospective cohort study

**DOI:** 10.1186/s12884-021-04146-0

**Published:** 2021-10-06

**Authors:** Dongqing Wang, Anne Marie Darling, Chloe R. McDonald, Nandita Perumal, Enju Liu, Molin Wang, Said Aboud, Willy Urassa, Andrea L. Conroy, Kyla T. Hayford, W. Conrad Liles, Kevin C. Kain, Wafaie W. Fawzi

**Affiliations:** 1 Department of Global Health and Population, Harvard T.H. Chan School of Public Health, Harvard University, Boston, MA 02120 USA; 2 Sandra Rotman Laboratories, University Health Network, Toronto, Ontario Canada; 3 Institutional Centers for Clinical and Translational Research, Boston Children’s Hospital, Boston, MA USA; 4 Department of Biostatistics, Harvard T.H. Chan School of Public Health, Harvard University, Boston, MA USA; 5 Department of Epidemiology, Harvard T.H. Chan School of Public Health, Harvard University, Boston, MA USA; 6 Department of Microbiology and Immunology, Muhimbili University of Health and Allied Sciences, Dar es Salaam, Tanzania; 7 Department of Pediatrics, Ryan White Center for Pediatric Infectious Disease and Global Health, Indiana University School of Medicine, Indianapolis, Indiana USA; 8 Department of International Health, Johns Hopkins Bloomberg School of Public Health, Baltimore, MD USA; 9 Department of Medicine, University of Washington, Seattle, Washington USA; 10 Department of Medicine, University of Toronto and University Health Network, Toronto, Ontario Canada; 11 Department of Nutrition, Harvard T.H. Chan School of Public Health, Harvard University, Boston, MA USA

**Keywords:** Pregnancy, Weight gain during pregnancy, Gestational weight, Inflammatory proteins, Metabolic proteins, Tanzania, Sub-Saharan Africa, Leptin, CH3L1, Biomarkers

## Abstract

**Background:**

Gestational weight gain (GWG) has critical implications for maternal and child health. Inflammation and angiogenesis are implicated in various aspects of maternal metabolism that may play a role in gestational weight gain. The associations of inflammatory, angiogenic, and metabolic pathways with GWG are yet to be elucidated. This study evaluated associations between a panel of inflammatory, angiogenic, and metabolic proteins measured in mid-pregnancy and gestational weight gain.

**Methods:**

Pregnant women were enrolled from Dar es Salaam, Tanzania, between 2001 and 2004. The participants were enrolled at mid-pregnancy (12 to 27 weeks of gestation) and followed up until delivery. This analysis focused on a cohort of 1002 women who were primigravid, had singleton live births, had longitudinal measures of gestational weight, and whose mid-pregnancy plasma samples underwent analysis for 18 proteins.

**Results:**

Higher plasma concentrations of leptin (mean difference in GWG percent adequacy comparing highest with lowest quartiles: 10.24; 95% CI 3.31, 17.16; *p*-trend = 0.003) and chitinase-3-like protein-1 (CH3L1) (mean difference in GWG percent adequacy comparing highest with lowest quartiles: 7.02; 95% CI 0.31, 13.72; *p*-trend = 0.007) were associated with greater GWG in a dose-response pattern. Higher leptin concentrations were associated with a lower risk of inadequate GWG (risk ratio comparing highest with lowest quartiles: 0.77; 95% CI 0.65, 0.91; *p*-trend = 0.001) and a higher risk of excessive GWG (risk ratio comparing highest with lowest quartiles: 1.57; 95% CI 1.03, 2.39; *p*-trend = 0.03). Higher CH3L1 concentrations were associated with a higher risk of excessive GWG (*p*-trend = 0.007). The associations of leptin and CH3L1 with inadequate GWG were stronger during the second than the third trimester. The other 16 proteins examined were not significantly associated with GWG.

**Conclusions:**

Mid-pregnancy plasma leptin concentrations may be associated with GWG and have clinical predictive utility in identifying women at a higher risk of inadequate or excessive gestational weight gain.

**Supplementary Information:**

The online version contains supplementary material available at 10.1186/s12884-021-04146-0.

## Background

Gestational weight gain (GWG) is a complex process that supports the functions of growth and development of the fetus during pregnancy [1], with important implications for maternal and child health. On the one hand, inadequate GWG increases the risks of low birthweight [1–3], preterm birth [1, 3, 4], small for gestational age [1, 2, 4], and fetal and neonatal death [5, 6]. On the other hand, excessive GWG is a risk factor for maternal morbidity [7], large for gestational age [1, 4] and postpartum weight retention [1, 2, 8]. Emerging evidence indicates that excessive GWG also increases the risk of the offspring being overweight or obese in their childhood and adulthood [8–12]. GWG is an essential target for antenatal monitoring [13] and preconceptional care [14]. In 2009, the National Academy of Medicine, formerly the Institute of Medicine (IOM), released guidelines on the recommended weight gain during pregnancy [1].

Accumulating evidence suggests that many women in low- and middle-income countries experience inadequate GWG. The mean level of GWG in sub-Saharan Africa has been estimated to be only 50 to 60% of the amount recommended by the IOM [15–17]. Pregnant women in the United Republic of Tanzania have a mean GWG level of 5.5 kg, which is less than 50% of the minimum recommendation (11.5 kg in normal-weight women and 12.5 kg in underweight women) and is among the lowest in sub-Saharan Africa [17]. At the same time, there is an emerging burden of excessive GWG in resource-constrained countries [16], which is likely to continue increasing with the nutritional transition to more Western diets, the increasingly sedentary lifestyles, and the rising tide of overweight and obesity [18, 19].

GWG is simultaneously affected by various maternal characteristics, including demographics, reproductive history, dietary intake, physical activity, psychological factors, and pre-existing health conditions [1]. Inflammatory and angiogenic processes and crosstalk between these pathways may also play a role in the accumulation of weight during pregnancy. However, the associations between inflammatory, angiogenic, and metabolic proteins and GWG remain poorly elucidated, especially in sub-Saharan Africa, where inflammation induced by common infections remains high and pregnant women are at a greater risk of poor birth outcomes [20].

Assessing the associations of inflammatory, angiogenic, and metabolic proteins with GWG may shed light on the mechanisms that contribute to GWG and reveal potential markers for antenatal monitoring of inadequate or excessive GWG. In this study, we evaluated associations of a panel of proteins measured in mid-pregnancy with GWG adequacy levels in Tanzania. We also aimed to explore whether potential associations may be modified by maternal characteristics.

## Methods

### Study design and study population

This study is a secondary analysis of a randomized, double-blind, placebo-controlled trial of maternal multiple micronutrient supplementation (MMS) in Tanzania. The primary aim of the study was to assess the impacts of prenatal MMS on fetal loss, preterm birth, and low birthweight. The design and findings of the parent study have been described elsewhere [21]. Briefly, pregnant women who attended antenatal clinics in Dar es Salaam, Tanzania were enrolled between August 2001 and July 2004. The eligibility criteria included: 1) negative for HIV infection based on HIV-1 serologic status [22]; 2) gestational age (GA) between 12 and 27 weeks at enrollment, based on the date of last menstrual period; 3) maternal age of 18 years or older; and 4) an intention to deliver and stay in Dar es Salaam for at least 1 year after delivery. From enrollment through delivery, the women were randomly assigned to receive daily oral MMS or placebo. The study enrolled 8428 eligible women, of whom 6 died before delivery and 43 were lost to follow-up by the time of delivery. Among the remaining 8379 women, 8223 were pregnant with singletons. This study was approved by the institutional review boards at Muhimbili University of Health and Allied Sciences, the University Health Network (Toronto, Ontario, Canada), and Harvard T.H. Chan School of Public Health. All women provided written informed consent to participate.

### Data collection

Participants had study visits by medical providers monthly until 32 weeks of gestation, then every 2 weeks until 36 weeks of gestation, and then weekly until 6 weeks after delivery. At baseline enrollment, research nurses used a questionnaire to collect data on demographic characteristics, socioeconomic status, reproductive and medical history, and behavioural factors, including smoking and alcohol consumption. Household ownership of five assets, including sofa or couch, television, radio, refrigerator, and fan, was collected at baseline using a questionnaire, A baseline wealth index was constructed based on household asset ownership using principal component analysis [23], and the same index has been used in previous publications of this cohort [24–27]. At baseline, research nurses measured maternal height to the nearest 0.1 cm using a stadiometer with headcovers and shoes removed. Weight was measured at baseline and every follow-up visit to the nearest 100 g using balanced scales with the participant wearing light clothing without shoes. Trained research assistants obtained information on dietary intake using monthly 24-h recalls until 36 weeks of gestation.

Maternal peripheral blood samples were collected in EDTA vacutainer tubes at enrollment. The plasma samples underwent analysis for 18 inflammatory, angiogenic, and metabolic proteins among a random subset of 1078 women who were primigravid, had singleton live births, and had stored plasma samples available [26]. The measurements of the proteins have been described elsewhere [20, 26]. Briefly, the 18 proteins included 1) angiopoietin-1 (Ang-1); 2) angiopoietin-2 (Ang-2); 3) angiopoietin-like 3 (Angptl3); 4) vascular endothelial growth factor (VEGF-A); 5) soluble fms-like tyrosine kinase 1 (sFlt-1); 6) soluble tumor necrosis factor receptor 2 (sTNFR2); 7) placental growth factor (PGF); 8) macrophage inflammatory protein-1 beta (MIPβ/CCL4); 9) monocyte chemoattractant protein-1 (MCP-1/CCL2); 10) leptin; 11) interleukin-1 beta (IL-1β); 12) interleukin-18 binding protein (IL-18 BP); 13) soluble intercellular adhesion molecule-1 (sICAM1); 14) complement factor D (Factor D); 15) soluble endoglin (sEng); 16) C-reactive protein (CRP); 17) chitinase-3-like protein-1 (CHI3L1); and 18) complement component C5a (C5a). We selected this panel of proteins based on previous literature and work in our laboratory indicating their associations with pregnancy physiology and adverse birth outcomes [28–34].

### Statistical analysis

#### Estimation of early-pregnancy weight

As the participants were 12 to 27 weeks of gestation at enrollment, 97.7% of the participants did not have an observed weight measure during the first trimester of pregnancy. An accurate assessment of GWG requires a pre-pregnancy weight measure or a measure of first-trimester weight as a proxy [35]. We have previously developed an approach to imputing first-trimester weight. The development and validation of this approach and the comparison with alternative strategies have been described in detail elsewhere [35]. Briefly, this approach employed mixed-effects models with restricted cubic splines to estimate the gestational weight in the first trimester using longitudinal weight measures collected later during pregnancy. We imputed the weight at 9^0/7^ weeks instead of another time point (e.g., pre-pregnancy weight) to avoid undue extrapolation. As weight gain during the first trimester is minimal [1], the weight imputed close to the midpoint of the first trimester served as a reasonable proxy for the pre-pregnancy weight.

First-trimester body mass index (BMI) was calculated by dividing first-trimester weight (observed during the first trimester or imputed at 9^0/7^ weeks) in kilograms by the square of height in meters. For women aged ≥19 years old, we used the cutoffs by the World Health Organization (WHO) to define underweight (BMI: < 18.5), normal weight (BMI: 18.5 to < 25.0), overweight (BMI: 25.0 to < 30.0), and obesity (BMI: ≥ 30.0). For adolescent women (i.e., less than 20 years old based on the United Nations’ definition), we used the WHO growth reference to define underweight (BMI-for-age Z-score: < − 2 SD), normal weight (BMI-for-age Z-score: − 2 SD to < 1 SD), overweight (BMI-for-age Z-score: 1 SD to < 2 SD), and obese (BMI-for-age Z-score: ≥ 2 SD) [36].

#### Metrics of gestational weight gain

GWG was quantified as the percent adequacy compared to the weight gain recommended by the IOM. First, we calculated the total GWG (in kilograms) for each participant as the difference between the last available weight during pregnancy and the first-trimester weight (imputed or observed). Second, we estimated the IOM-recommended GWG (in kilograms) for each woman at the time when the last weight measure was taken. Finally, the GWG percent adequacy was calculated by dividing the observed GWG by the recommended GWG at the last weight measurement. The overall formula for the GWG percent adequacy is displayed below.

$$\mathrm{GWG}\;\mathrm{percent}\;\mathrm{adequacy}\;\left(\%\right)\;=\frac{\mathrm{Observed}\;\mathrm{GWG}}{\mathrm{Recommended}\;\mathrm{GWG}}\times100=\frac{\mathrm{last}\;\mathrm{available}\;\mathrm{weight}-\mathrm{imputed}\;\mathrm T1\;\mathrm{weight}}{\mathrm{expected}\;\mathrm T1\;\mathrm{GWG}\;+\;\left(\mathrm{GA}\;\mathrm{at}\;\mathrm{last}\;\mathrm{available}\;\mathrm{weight}\;-13.86\;\mathrm{wks}\right)\;\times\;\mathrm{recommended}\;\mathrm{weekly}\;\mathrm{rate}\;\mathrm{of}\;\mathrm{GWG}\;\mathrm{for}\;\mathrm T2\;\&\;\mathrm T3}\times100$$

The GWG percent adequacy has been calculated in the same way in previous studies [37]. This metric accounts for the different gestational durations at last weight measurements and takes advantage of well-established recommendations. In addition to the continuous measure of percent adequacy, we created binary outcomes that correspond to inadequate, adequate, and excessive GWG. We defined inadequate GWG as percent adequacy < 90%, adequate GWG as percent adequacy of 90 to 125%, and excessive GWG as percent adequacy > 125%, consistent with previous literature [37]. We also calculated the trimester-specific weekly rates of weight gain using the first and last available measures within the second and third trimesters; inadequate, adequate, and excessive weekly GWG were defined similarly based on the rate recommended by the IOM [1].

#### Regression analyses

The regression analyses included 1002 primigravid women with singleton live births who had data available on plasma concentrations of the proteins (which was measured in a random subset of all women with singleton live births), longitudinal gestational weight measures, and key potential confounders (education level, marital status, and wealth index). The plasma concentrations of each protein were divided into quartiles based on the distributions in the analytical sample. We used linear models to evaluate the associations of the proteins with the continuous metric of GWG percent adequacy and reported the mean differences and 95% confidence intervals (CIs), comparing each quartile to the lowest quartile. We used log-binomial models to evaluate the associations of the proteins with the binary metrics of GWG, including inadequate GWG and excessive GWG. We used modified Poisson models with robust variance estimation to handle model convergence issues whenever necessary [38, 39]. We reported the risk ratios (RRs) and 95% CIs comparing each quartile to the lowest quartile.

All models were adjusted for potential confounders, including maternal age at enrollment, maternal education level, marital status, maternal occupation, household wealth index, total energy intake, and first-trimester BMI category. We also adjusted for the randomly assigned intervention (MMS or control) as prenatal MMS has been previously shown to increase GWG in this cohort of women [27].

For proteins associated with GWG in the overall sample, we additionally included cross-product terms and conducted subgroup analyses to explore effect modification by the following four maternal characteristics: 1) prenatal regimen (MMS or control); 2) first-trimester BMI category (underweight, normal-weight, or overweight/obese); 3) maternal anaemia status at enrollment defined as any anaemia (haemoglobin < 11 g/dL) or no anaemia [40]; and 4) maternal stature (< 150 cm or ≥ 150 cm). All analyses were conducted using SAS 9.4 (SAS Institute Inc., Cary, North Carolina) with a two-sided *α* level of 0.05. We did not adjust for multiple testing [41].

## Results

General characteristics at enrollment and GWG outcomes of the pregnant women are presented in Table 1, and their mid-pregnancy concentrations of the proteins are presented in Table 2. At baseline, the participants were on average 22 years (range: 18 to 41 years) of age and at 21 weeks (range: 12 to 27 weeks) of gestation. All participants included in this analysis were primigravida and pregnant with a singleton fetus. Over 90% of the sample had 5 or more years of education, nearly 79% were married or cohabiting, and approximately 78% were unemployed. Approximately 12, 75, 11, and 2% of the participants were underweight, normal-weight, overweight, and obese, respectively; the mean BMI during early pregnancy was 21.8 kg/m^2^ (SD: 3.27 kg/m^2^). The median GWG percent adequacy was 88.3% (25th percentile, 75th percentile: 68.8, 112.3%). Approximately 53, 30, and 17% of the participants had inadequate, adequate, and excessive GWG, respectively.

**Table 1:** Maternal characteristics at enrollment and gestational weight gain outcomes in a cohort of pregnant women in Dar es Salaam, Tanzania, 2001-2004^a^

Maternal characteristics	
Number of women, *N*	1002
Age at enrollment, years	21.8 (3.17)
Gestational age at enrollment, weeks	21.3 (3.55)
Maternal education in years, %	
0 to 4	83 (8.3)
5 to 7	659 (65.8)
8 to 11	214 (21.4)
≥ 12	46 (4.6)
Marital status, %	
Married/cohabiting	789 (78.7)
Single/divorced/widowed	213 (21.3)
Maternal occupation,^b^ %	
Unemployed	762 (78.5)
Employed	209 (21.5)
First-trimester BMI^c^	21.8 (3.3)
First-trimester BMI category,^c^ %	
Underweight	120 (12.0)
Normal weight	749 (74.8)
Overweight	115 (11.5)
Obese	18 (1.8)
Total energy intake,^d^ kcal/d	2278.0 (840.6)
Intervention assignment, %	
Multiple micronutrient supplementation	523 (52.2)
Control	479 (47.8)
**Gestational weight gain**	
Gestational weight gain percent adequacy	88.3 (68.8, 112.3)
Category of gestational weight gain adequacy,^e^ %	
Inadequate	533 (53.2)
Adequate	299 (29.8)
Excessive	170 (17.0)

^a^ Values are mean (standard deviation) for normally distributed continuous variables, median (25th percentile, 75th percentile) for continuous variables with skewed distributions, and count (percentage) for categorical variables. BMI, body mass index

^b^ Maternal occupation was missing for 31 women

^c^ Based on the observed weight during the first trimester or imputed weight at 9^0/7^ weeks

^d^ Total energy intake was calculated as the average intake during pregnancy based on multiple 24-h recalls; missing for 61 women

^e^ Inadequate, adequate, and excessive gestational weight gain were defined as < 90, 90 to 125%, and > 125% percent adequacy, respectively, based on the Institute of Medicine guidelines

**Table 2:** Mid-pregnancy plasma concentrations of inflammatory, angiogenic, and metabolic proteins in a cohort of pregnant women in Dar es Salaam, Tanzania, 2001-2004^a^

Proteins	***N***	Median (25th percentile, 75th percentile)
Ang-1, ng/mL	1001	14.88 (8.15, 23.57)
Ang-2, ng/mL	1001	4.63 (2.01, 9.21)
Angptl3, ng/mL	996	87.37 (56.49, 130.85)
VEGF-A, pg/mL	998	39.66 (7.81, 262.25)
sFlt-1, ng/mL	967	1.27 (0.55, 3.03)
sTNFR2, ng/mL	999	5.04 (3.27, 7.50)
PGF, ng/mL	950	1.18 (0.54, 2.24)
MIPβ/CCL4, pg/mL	982	147.21 (56.10, 346.06)
MCP-1/CCL2, pg/mL	990	75.29 (7.81, 868.80)
Leptin, ng/mL	1001	8.39 (4.66, 13.79)
IL-1β, pg/mL	386	20.84 (3.91, 76.40)
IL-18 BP, ng/mL	1002	12.98 (8.74, 19.03)
sICAM1, ng/mL	1002	151.39 (103.53, 226.81)
Factor D, ng/mL	377	487.32 (326.87, 681.80)
sEng, ng/mL	1001	21.19 (14.62, 28.10)
CRP, *μ* g/mL	372	1.87 (0.81, 4.14)
CHI3L1, ng/mL	1002	37.54 (22.75, 66.69)
C5a, ng/mL	991	79.20 (30.65, 236.78)

^a^ *Ang-1* angiopoietin-1; *Ang-2* angiopoietin-2; *Angptl3* angiopoietin-like 3; *C5a* complement component C5a; *CHI3L1* chitinase-3-like protein-1; *CRP* C-reactive protein; *Factor D* complement factor D; *IL-18 BP* interleukin-18 binding protein; *IL-1β* interleukin-1 beta; *MCP-1/CCL2* monocyte chemoattractant protein-1; *MIPβ/CCL4* macrophage inflammatory protein-1 beta; *PGF* placental growth factor; *sEng* soluble endoglin; *sFlt-1* soluble fms-like tyrosine kinase 1; *sICAM1* soluble intercellular adhesion molecule-1; *sTNFR2* soluble tumor necrosis factor receptor 2; *VEGF-A* vascular endothelial growth factor

Higher mid-pregnancy plasma leptin concentrations were associated with a greater GWG percent adequacy (Table 3). Participants in the third and fourth quartiles of leptin concentrations had 6.9% (95% CI 0.12, 13.66) and 10.2% (95% CI 3.31, 17.16) greater GWG percent adequacy, respectively, compared with those in the lowest quartile (*p*-trend = 0.003). Higher mid-pregnancy plasma CHI3L1 concentrations were also associated with a greater GWG percent adequacy. Participants in the highest quartile of CHI3L1 had 7.0% (95% CI 0.31, 13.72) greater GWG percent adequacy compared with women in the lowest quartile (*p*-trend = 0.007). The other 16 proteins examined were not associated with GWG percent adequacy.

**Table 3:** Mid-pregnancy plasma concentrations of inflammatory, angiogenic, and metabolic proteins and gestational weight gain percent adequacy in a cohort of pregnant women in Dar es Salaam, Tanzania, 2001-2004^a,b^

	Quartile 1		Quartile 2		Quartile 3		Quartile 4		***P***-trend^**c**^
	*n*	Mean difference (95% CI)	*n*	Mean difference (95% CI)	*n*	Mean difference (95% CI)	*n*	Mean difference (95% CI)	
Ang-1	250	0.00 (Reference)	250	6.30 (−0.44, 13.03)	251	1.47 (−5.22, 8.16)	250	4.88 (−1.86, 11.62)	0.4
Ang-2	250	0.00 (Reference)	250	−3.47 (−10.16, 3.21)	251	−2.25 (−8.95, 4.44)	250	−0.76 (−7.49, 5.98)	0.9
Angptl3	249	0.00 (Reference)	249	2.36 (−4.33, 9.05)	249	0.98 (−5.71, 7.67)	249	1.20 (−5.51, 7.91)	0.9
VEGF-A	235	0.00 (Reference)	263	−6.52 (−13.23, 0.18)	251	−4.01 (−10.84, 2.82)	249	−3.02 (−9.80, 3.76)	0.9
sFlt-1	241	0.00 (Reference)	242	−0.60 (−6.13, 7.33)	242	0.87 (−5.89, 7.63)	242	1.31 (−5.44, 8.05)	0.7
sTNFR2	249	0.00 (Reference)	250	−2.51 (−9.23, 4.22)	250	−0.36 (−7.06, 6.34)	250	−3.73 (−10.46, 3.00)	0.4
PGF	237	0.00 (Reference)	238	−4.24 (−11.01, 2.53)	238	−1.84 (−8.64, 4.95)	237	0.60 (−6.24, 7.45)	0.5
MIPβ/CCL4	245	0.00 (Reference)	246	0.34 (−6.43, 7.11)	246	−0.85 (−7.61, 5.91)	245	0.05 (−6.77, 6.87)	1.0
MCP-1/CCL2	247	0.00 (Reference)	248	−1.73 (−8.48, 5.03)	248	−2.74 (−9.46, 3.97)	247	−1.60 (−8.36, 5.15)	0.9
Leptin	249	0.00 (Reference)	251	3.45 (−3.23, 10.12)	251	6.89 (0.12, 13.66)	250	10.24 (3.31, 17.16)	0.003
IL-1β	105	0.00 (Reference)	88	4.45 (−6.72, 15.63)	97	5.46 (−5.60, 16.52)	96	−4.20 (−15.19, 6.79)	0.2
IL-18 BP	250	0.00 (Reference)	251	−4.60 (−11.28, 2.08)	251	−2.79 (−9.49, 3.90)	250	−4.85 (− 11.56, 1.87)	0.3
sICAM1	250	0.00 (Reference)	250	−4.45 (−11.16, 2.26)	252	−4.87 (−11.57, 1.83)	250	−3.66 (− 10.35, 3.03)	0.4
Factor D	94	0.00 (Reference)	94	−1.71 (− 13.04, 9.61)	95	5.88 (−5.46, 17.22)	94	3.70 (−7.93, 15.32)	0.4
sEng	250	0.00 (Reference)	250	3.62 (−3.04, 10.29)	251	−0.10 (−6.78, 6.58)	250	2.62 (−4.10, 9.33)	0.7
CRP	93	0.00 (Reference)	93	−2.76 (−14.16, 8.64)	93	−5.67 (−16.95, 5.61)	93	−0.94 (− 12.34, 10.45)	0.9
CHI3L1	250	0.00 (Reference)	251	−3.00 (−9.65, 3.65)	251	−0.73 (−7.40, 5.93)	250	7.02 (0.31, 13.72)	0.007
C5a	247	0.00 (Reference)	248	2.01 (−4.66, 8.67)	248	4.27 (−2.40, 10.94)	248	2.51 (−4.17, 9.19)	0.7

^a^ Estimates were obtained from linear models comparing the upper three quartiles to the lowest quartile. *Ang-1* angiopoietin-1; *Ang-2* angiopoietin-2; *Angptl3* angiopoietin-like 3; *C5a* complement component C5a; *CHI3L1* chitinase-3-like protein-1; *CI* confidence interval; *CRP* C-reactive protein; *Factor D* complement factor D; *IL-18 BP* interleukin-18 binding protein; *IL-1β* interleukin-1 beta; *MCP-1/CCL2* monocyte chemoattractant protein-1; *MIPβ/CCL4* macrophage inflammatory protein-1 beta; *PGF* placental growth factor; *sEng* soluble endoglin; *sFlt-1* soluble fms-like tyrosine kinase 1; *sICAM1* soluble intercellular adhesion molecule-1; *sTNFR2* soluble tumor necrosis factor receptor 2; *VEGF-A* vascular endothelial growth factor

^b^ All models were adjusted for maternal age at enrollment (years), maternal education level (0 to 4 years, 5 to 7 years, 8 to 11 years, and ≥ 12 years), marital status (married or not), maternal occupation (employed or not), household wealth index (quintiles), total energy intake (kcal/d), intervention assignment (multiple micronutrient supplementation or control), and first-trimester BMI category (underweight, normal-weight, or overweight/obese). Missing data on maternal occupation and total energy intake were accounted for by using the missing indicator method

^c^ Computed by assigning the median concentration of each quartile to participants in the corresponding quartile as a continuous variable

Higher mid-pregnancy plasma leptin concentrations were associated with a lower risk of inadequate GWG and a higher risk of excessive GWG (Table 4). Participants in the highest quartile of leptin had 23% (RR = 0.77; 95% CI 0.65, 0.91) lower risk of inadequate GWG compared with women in the lowest quartile (*p*-trend = 0.001). Participants in the highest quartile of leptin had a 57% (RR = 1.57; 95% CI 1.03, 2.39) higher risk of excessive GWG compared with women in the lowest quartile (*p*-trend = 0.03). Higher mid-pregnancy plasma CHI3L1 concentrations were associated with a higher risk of excessive GWG (*p*-trend = 0.007), although none of the quartiles reached statistical significance. Analyses on the trimester-specific weekly rate of GWG showed that the associations of leptin and CHI3L1 with inadequate GWG were stronger during the second trimester than during the third trimester (Additional file 1). The other 16 proteins examined were not associated with inadequate or excessive GWG (Additional file 2).

**Table 4:** Mid-pregnancy plasma concentrations of leptin and chitinase-3-like protein-1 and inadequate and excessive gestational weight gain in a cohort of pregnant women in Dar es Salaam, Tanzania, 2001-2004^a,b^

	Inadequate GWG (***n*** = 533)	Excessive GWG (***n*** = 170)
	RR (95% CI)	RR (95% CI)
Leptin		
Quartile 1	1.00 (Reference)	1.00 (Reference)
Quartile 2	0.98 (0.85, 1.11)	1.24 (0.77, 2.00)
Quartile 3	0.90 (0.78, 1.04)	1.27 (0.82, 1.97)
Quartile 4	0.77 (0.65, 0.91)	1.57 (1.03, 2.39)
*P*-trend^c^	0.001	0.03
CHI3L1		
Quartile 1	1.00 (Reference)	1.00 (Reference)
Quartile 2	1.06 (0.91, 1.23)	0.85 (0.57, 1.28)
Quartile 3	0.95 (0.81, 1.12)	1.05 (0.73, 1.51)
Quartile 4	1.00 (0.85, 1.17)	1.35 (0.99, 1.84)
*P*-trend^c^	0.7	0.007

^a^ Estimates were obtained from log-binomial models. Modified Poisson models with robust variance estimation were used to handle model convergence issues whenever necessary. Inadequate and excessive gestational weight gain was defined as < 90% and > 125% percent adequacy, respectively, based on the Institute of Medicine guidelines. The reference outcome for inadequate gestational weight gain included adequate and excessive gestational weight gain; the reference outcome for excessive gestational weight gain included adequate and inadequate gestational weight gain. *CHI3L1* chitinase-3-like protein-1; *CI* confidence interval; *GWG* gestational weight gain; *RR* risk ratio

^b^ All models were adjusted for maternal age at enrollment (years), maternal education level (0 to 4 years, 5 to 7 years, 8 to 11 years, and ≥ 12 years), marital status (married or not), maternal occupation (employed or not), household wealth index (quintiles), total energy intake (kcal/d), intervention assignment (multiple micronutrient supplementation or control), and first-trimester BMI category (underweight, normal-weight, or overweight/obese). Missing data on maternal occupation and total energy intake were accounted for by using the missing indicator method

^c^ Computed by assigning the median concentration of each quartile to participants in the corresponding quartile as a continuous variable

Results from the exploratory analyses of interaction using cross-product terms are shown in Additional file 3. There were statistical interactions between leptin and first-trimester BMI category on GWG percent adequacy (p-interaction = 0.02), between leptin and baseline maternal anaemia on inadequate GWG (p-interaction = 0.04), and between leptin and maternal stature on excessive GWG (p-interaction = 0.02). There were also statistical interactions between CHI3L1 and first-trimester BMI category on GWG percent adequacy (p-interaction < 0.001) and between CHI3L1 and first-trimester BMI category on inadequate GWG (p-interaction = 0.001). Subgroup analyses of potential effect modification (Additional files 4 and 5) show that: 1) the positive associations of leptin and CHI3L1 with GWG percent adequacy were stronger among overweight or obese women than normal-weight and underweight women; 2) the inverse association of leptin with inadequate GWG was stronger among anaemic women than non-anaemic women; and 3) the inverse association of CHI3L1 with inadequate GWG was stronger among underweight women than normal-weight women.

## Discussion

In this observational analysis using data from a randomized controlled trial in Dar es Salaam, Tanzania, we report that higher mid-pregnancy plasma concentrations of leptin and CHI3L1 are associated with greater GWG percent adequacy in a dose-response pattern. We also find that higher mid-pregnancy plasma leptin concentrations are associated with a lower risk of inadequate GWG, while higher mid-pregnancy leptin and CHI3L1 concentrations are associated with a higher risk of excessive GWG. The associations of leptin and CHI3L1 with inadequate GWG are stronger during the second trimester than during the third trimester.

Inflammatory and angiogenic processes are involved in various aspects of maternal metabolism that may play a role in the accumulation of gestational weight. The IOM identifies the evaluation of whether maternal biomarkers can enhance clinical prediction and guide interventions for women with GWG outside the recommended ranges as an important research area [1]. However, the associations of inflammatory, angiogenic, and metabolic pathways with GWG are yet to be fully elucidated, especially in sub-Saharan Africa, which has a high burden of inadequate GWG and an emerging burden of excessive GWG [16, 17]. This study fills this gap by examining the associations between the mid-pregnancy plasma concentrations of a wide array of potential biomarkers and GWG.

Leptin is an adipocytokine synthesized by adipose tissues and the placenta during pregnancy [42]. In the first trimester, the placenta produces large amounts of leptin transferred primarily into the maternal circulation, resulting in a marked increase in maternal plasma leptin concentrations [42, 43]. Maternal plasma leptin concentrations peak in the late second or early third trimester and stay elevated throughout the remainder of the pregnancy [44]. Leptin is also implicated in various aspects of maternal metabolism, including satiety and energy balance, trophoblast invasion (vital for the establishment of pregnancy), placental growth, nutrient transfer, and the regulation of neuroendocrine functions [45–47]. Further, leptin is involved in immune response and T-cell activation and has pro-inflammatory properties associated with adverse pregnancy outcomes, including preeclampsia, gestational diabetes, preterm birth, and intrauterine growth restriction [20, 26, 42].

Findings from this study add to the emerging body of literature in high-income settings suggesting a positive association between mid-pregnancy leptin and GWG among women [48–50]. Notably, in a recent study among Canadian women, higher second-trimester leptin concentrations were associated with greater late-pregnancy GWG [50]. The positive association between leptin and weight during pregnancy is in contrast to leptin’s physiologic effects of satiety (inhibiting hunger) and diminishing fat storage among non-pregnant individuals [50]. Although there is an increased concentration of leptin in circulation during pregnancy, a positive energy balance is achieved through a decreased production of central leptin in adipose tissue and resistance to leptin’s central satiety effect [51, 52]. This inverse association between leptin and inadequate GWG is also in line with the inverse association between leptin and small for gestational age previously reported in the same cohort [20], as inadequate GWG has been consistently associated with a higher risk of small for gestational age [1].

The specific biological pathways through which leptin affects GWG remain to be evaluated. Leptin may contribute to a greater accumulation of maternal body fat and placental weight during pregnancy [42, 53]. Leptin may also represent a proxy measure of maternal insulin sensitivity, fat metabolism, or other mechanisms involved in weight gain, even if there are no direct mechanisms relating leptin per se to GWG [1, 48, 54]. Still, the consistent and strong association between mid-pregnancy leptin and late-pregnancy GWG suggests that leptin concentrations measured during mid-pregnancy may have predictive utility for the early identification of women at a higher risk of inadequate or excessive GWG, which should be evaluated in further prospective studies.

We conducted subgroup analyses to assess whether the associations of leptin and CHI3L1 with GWG may be modified by a few maternal characteristics. One notable finding is that the positive associations of leptin and CHI3L1 with GWG were stronger among overweight or obese women than underweight and normal-weight women. A previous study in Canada also finds that the positive association between second-trimester leptin concentrations and GWG is stronger in overweight women [50]. One potential explanation may be that the central satiety effect of leptin during pregnancy is weaker among overweight women [43, 55]. Others have suggested the presence of a positive feed-forward loop that enhances the weight-increasing effect of leptin during pregnancy among overweight women [50]. It should be noted, however, that these secondary analyses on effect modification are exploratory, and any statistical interaction must be interpreted with caution. Future studies with a more substantial sample size within each BMI category (especially obesity which was rare in our study) should specifically evaluate this effect modification of leptin by early-pregnancy BMI.

CHI3L1, also known as YKL-40 or cartilage glycoprotein-39, is a glycoprotein involved in inflammation and extracellular matrix remodeling [56, 57]. We found that higher mid-pregnancy plasma CHI3L1 concentrations were associated with greater weight gain during pregnancy, albeit to a smaller extent than leptin. Higher maternal plasma concentrations of CHI3L1 have been associated with a higher risk of spontaneous preterm birth in the same cohort of pregnant women [26]. However, the physiological implications of CHI3L1 in human pregnancy remain poorly understood. Future research is needed to better elucidate the role of CHI3L1 in pregnancy and perinatal outcomes.

This study provides evidence for the potential utility of biomarkers in the identification of pregnant women at high risks of gaining inadequate or excessive weight. Future work that seeks to provide early predictions of GWG may consider the values of biomarkers, especially leptin, as predictors. This study was conducted nearly two decades ago, and the prevalences of inadequate and excessive GWG may not accurately reflect the current burdens of suboptimal GWG. However, recent evidence shows that GWG in sub-Saharan Africa is extremely low and is not showing signs of improvements from the beginning of the new millennium [58]. The associations between biomarkers and GWG is unlikely to be heavily time-dependent, and our findings remain timely for future investigations into biomarkers of GWG.

The strengths of this study include the prospective design, the inclusion of an extensive panel of proteins, the use of GWG percent adequacy measures that are independent of gestational duration, and the focus on an understudied population in sub-Saharan Africa. We maintained an *α* level of 0.05 and did not account for the examination of numerous proteins or outcomes. This may have increased the probability of type I error for the composite null hypothesis that “none of the proteins were associated with GWG.” However, the aim of the study was to discover potentially important biomarkers for further investigation, so quantitative adjustments for multiple testing (e.g., the Bonferroni correction) would have inflated the probability of type II error and masked potential associations [41].

This study has some potential limitations. First, pre-pregnancy weight was not available in this study, and for most (some 98%) of the participants, we imputed first-trimester weight at 9 weeks as a proxy for pre-pregnancy weight. This may have introduced measurement errors in the GWG metrics. However, this methodological approach to imputing early-pregnancy weight based on weight measures collected later during pregnancy has previously been validated using pregnant women in Dar es Salaam, Tanzania, with good imputation accuracy [35], and any misclassification is likely nondifferential with respect to concentrations of the proteins. Second, gestational age in this study was calculated based on self-reported last menstrual period (as opposed to the gold standard of ultrasound, which was difficult to access at the time of the study), which may have introduced measurement error in gestational dating. Third, as an observational analysis, the potential of residual confounding could not be ruled out, even though we adjusted extensively for potential confounders. Finally, the proteins were measured only once (during mid-pregnancy). Future studies with repeatedly collected measures of proteins should evaluate the longitudinal associations between these proteins and GWG metrics over the course of pregnancy.

## Conclusions

Leptin and potentially CHI3L1 may be associated with a greater weight gain during pregnancy among pregnant women in sub-Saharan Africa. Future studies should examine the physiological roles of these proteins in the accumulation of weight during pregnancy. Further work is also needed to evaluate the clinical predictive utility of mid-pregnancy maternal leptin concentrations in the early identification of women at high risks of inadequate and excessive GWG.

## Supplementary Information

**Additional file 1:.**

**Additional file 2:.**

**Additional file 3:.**

**Additional file 4:.**

**Additional file 5:.**

## Data Availability

The datasets generated and/or analysed during the current study are not publicly available due to the need to protect the privacy of individuals that participated in the study, but are available from the corresponding author on reasonable request.

## Abbreviations

Ang-1: Angiopoietin-1
Ang-2: Angiopoietin-2
Angptl3: Angiopoietin-like 3
BMI: Body mass index
C5a: Complement component C5a
CHI3L1: Chitinase-3-like protein-1
CRP: C-reactive protein
Factor D: Complement factor D
GA: Gestational age
GWG: Gestational weight gain
IL-18 BP: Interleukin-18 binding protein
IL1β: Interleukin-1 beta
IOM: Institute of Medicine
MCP-1/CCL2: Monocyte chemoattractant protein-1
MIPβ/CCL4: Macrophage inflammatory protein-1 beta
MMS: Multiple micronutrient supplementation
MUAC: Mid-upper arm circumference
PGF: Placental growth factor
RCT: Randomized controlled trial
sEng: Soluble endoglin
sFlt-1: Soluble fms-like tyrosine kinase 1
sICAM1: Soluble intercellular adhesion molecule-1
sTNFR2: Soluble tumor necrosis factor receptor 2
T1: First trimester
T2: Second trimester
T3: Third trimester
VEGF-A: Vascular endothelial growth factor
