# Supplementary material for: Plasma concentrations of leptin at mid-pregnancy are associated with gestational weight gain among pregnant women in Tanzania: a prospective cohort study
Source: BMC Pregnancy Childbirth. 2021 Oct 6;21:675. doi: 10.1186/s12884-021-04146-0 (PMC8495974; doi:10.1186/s12884-021-04146-0)
Supplement: Supplementary file 1 — Additional file 1:. [file 12884_2021_4146_MOESM1_ESM.docx]

**Additional file 1**  Mid-pregnancy plasma concentrations of leptin and chitinase-3-like protein-1 and trimester-specific weekly rate of gestational weight gain in a cohort of pregnant women in Dar es Salaam, Tanzania, 2001-2004^a,b^

|  | **Inadequate weekly rate of GWG** | | **Excessive weekly rate of GWG** | |
| --- | --- | --- | --- | --- |
|  | RR (95% CI) | | RR (95% CI) | |
|  | Second trimester | Third trimester | Second trimester | Third trimester |
| Leptin |  |  |  |  |
| Quartile 1 | 1.00 (Reference) | 1.00 (Reference) | 1.00 (Reference) | 1.00 (Reference) |
| Quartile 2 | 0.97 (0.81, 1.18) | 0.93 (0.79, 1.09) | 0.91 (0.63, 1.30) | 0.93 (0.66, 1.31) |
| Quartile 3 | 0.84 (0.69, 1.03) | 0.87 (0.73, 1.05) | 1.17 (0.84, 1.62) | 1.17 (0.85, 1.61) |
| Quartile 4 | 0.75 (0.60, 0.95) | 0.85 (0.70, 1.02) | 1.21 (0.88, 1.68) | 1.21 (0.89, 1.65) |
| *P*-trend^c^ | 0.008 | 0.09 | 0.1 | 0.1 |
| CHI3L1 |  |  |  |  |
| Quartile 1 | 1.00 (Reference) | 1.00 (Reference) | 1.00 (Reference) | 1.00 (Reference) |
| Quartile 2 | 0.98 (0.81, 1.18) | 0.89 (0.75, 1.06) | 1.18 (0.85, 1.63) | 0.96 (0.70, 1.30) |
| Quartile 3 | 0.87 (0.71, 1.06) | 0.88 (0.74, 1.05) | 1.05 (0.74, 1.47) | 1.20 (0.90, 1.59) |
| Quartile 4 | 0.79 (0.64, 0.99) | 0.88 (0.74, 1.05) | 1.36 (0.99, 1.86) | 1.16 (0.87, 1.54) |
| *P*-trend^c^ | 0.03 | 0.3 | 0.08 | 0.2 |

^a^ Estimates were obtained from log-binomial models. Modified Poisson models with robust variance estimation were used to handle model convergence issues whenever necessary. Trimester-specific weekly rates of weight gain were calculated using the first and last available measures within each trimester. Inadequate and excessive were defined as < 90% and > 125% of the recommended weekly rate of weight gain, respectively, based on the Institute of Medicine guidelines. CHI3L1, chitinase-3-like protein-1; CI, confidence interval; GWG, gestational weight gain; RR, risk ratio.

^b^ All models were adjusted for maternal age at enrollment (years), maternal education level (0 to 4 years, 5 to 7 years, 8 to 11 years, and $\geq$ 12 years), marital status (married or not), maternal occupation (employed or not), household wealth index (quintiles), total energy intake (kcal/d), intervention assignment (multiple micronutrient supplementation or control), and first-trimester BMI category (underweight, normal-weight, or overweight/obese). Missing data on maternal occupation and total energy intake were accounted for by using the missing indicator method.

^c^ Computed by assigning the median concentration of each quartile to participants in the corresponding quartile as a continuous variable.
