# Supplementary material for: Plasma concentrations of leptin at mid-pregnancy are associated with gestational weight gain among pregnant women in Tanzania: a prospective cohort study
Source: BMC Pregnancy Childbirth. 2021 Oct 6;21:675. doi: 10.1186/s12884-021-04146-0 (PMC8495974; doi:10.1186/s12884-021-04146-0)
Supplement: Supplementary file 2 — Additional file 2:. [file 12884_2021_4146_MOESM2_ESM.docx]

**Additional file 2** Mid-pregnancy plasma concentrations of inflammatory, angiogenic, and metabolic proteins and inadequate and excessive gestational weight gain in a cohort of pregnant women in Dar es Salaam, Tanzania, 2001-2004^a,b^

|  | **Inadequate GWG**  **(*n* = 533)** | **Excessive GWG**  **(*n* = 170)** |
| --- | --- | --- |
|  | RR (95% CI) | RR (95% CI) |
| Ang-1 |  |  |
| Quartile 1 | 1.00 (Reference) | 1.00 (Reference) |
| Quartile 2 | 1.09 (0.93, 1.27) | 1.10 (0.77, 1.58) |
| Quartile 3 | 1.06 (0.91, 1.23) | 0.89 (0.63, 1.27) |
| Quartile 4 | 0.98 (0.83, 1.17) | 1.10 (0.78, 1.55) |
| *P*-trend^c^ | 0.7 | 0.8 |
| Ang-2 |  |  |
| Quartile 1 | 1.00 (Reference) | 1.00 (Reference) |
| Quartile 2 | 1.00 (0.86, 1.16) | 0.99 (0.71, 1.39) |
| Quartile 3 | 0.94 (0.80, 1.10) | 0.96 (0.68, 1.36) |
| Quartile 4 | 0.93 (0.80, 1.08) | 1.05 (0.76, 1.46) |
| *P*-trend^c^ | 0.3 | 0.7 |
| Angptl3 |  |  |
| Quartile 1 | 1.00 (Reference) | 1.00 (Reference) |
| Quartile 2 | 0.99 (0.85, 1.15) | 0.88 (0.61, 1.26) |
| Quartile 3 | 0.92 (0.79, 1.08) | 0.88 (0.61, 1.26) |
| Quartile 4 | 1.00 (0.86, 1.16) | 1.12 (0.81, 1.56) |
| *P*-trend^c^ | 0.9 | 0.3 |
| VEGF-A |  |  |
| Quartile 1 | 1.00 (Reference) | 1.00 (Reference) |
| Quartile 2 | 1.10 (0.94, 1.29) | 0.79 (0.55, 1.14) |
| Quartile 3 | 1.07 (0.91, 1.25) | 0.93 (0.65, 1.34) |
| Quartile 4 | 1.13 (0.97, 1.32) | 1.13 (0.80, 1.59) |
| *P*-trend^c^ | 0.3 | 0.1 |
| sFlt-1 |  |  |
| Quartile 1 | 1.00 (Reference) | 1.00 (Reference) |
| Quartile 2 | 0.92 (0.79, 1.09) | 0.94 (0.64, 1.37) |
| Quartile 3 | 1.00 (0.85, 1.17) | 1.14 (0.81, 1.62) |
| Quartile 4 | 0.98 (0.84, 1.15) | 1.23 (0.87, 1.75) |
| *P*-trend^c^ | 0.8 | 0.1 |
| sTNFR2 |  |  |
| Quartile 1 | 1.00 (Reference) | 1.00 (Reference) |
| Quartile 2 | 1.10 (0.94, 1.28) | 0.86 (0.59, 1.25) |
| Quartile 3 | 1.06 (0.90, 1.24) | 0.95 (0.68, 1.33) |
| Quartile 4 | 1.10 (0.95, 1.29) | 0.89 (0.64, 1.24) |
| *P*-trend^c^ | 0.3 | 0.6 |
| PGF |  |  |
| Quartile 1 | 1.00 (Reference) | 1.00 (Reference) |
| Quartile 2 | 1.05 (0.90, 1.23) | 0.89 (0.63, 1.26) |
| Quartile 3 | 1.07 (0.92, 1.25) | 0.88 (0.63, 1.23) |
| Quartile 4 | 1.02 (0.87, 1.20) | 1.22 (0.86, 1.72) |
| *P*-trend^c^ | 1.0 | 0.1 |
| MIPβ/CCL4 |  |  |
| Quartile 1 | 1.00 (Reference) | 1.00 (Reference) |
| Quartile 2 | 0.94 (0.81, 1.10) | 1.01 (0.68, 1.50) |
| Quartile 3 | 0.95 (0.81, 1.11) | 0.95 (0.66, 1.36) |
| Quartile 4 | 0.98 (0.84, 1.13) | 1.17 (0.82, 1.65) |
| *P*-trend^c^ | 0.9 | 0.3 |
| MCP-1/CCL2 |  |  |
| Quartile 1 | 1.00 (Reference) | 1.00 (Reference) |
| Quartile 2 | 1.06 (0.90, 1.24) | 0.78 (0.54, 1.14) |
| Quartile 3 | 1.10 (0.95, 1.28) | 1.01 (0.72, 1.41) |
| Quartile 4 | 1.07 (0.91, 1.25) | 1.08 (0.76, 1.53) |
| *P*-trend^c^ | 0.8 | 0.2 |
| IL-1β |  |  |
| Quartile 1 | 1.00 (Reference) | 1.00 (Reference) |
| Quartile 2 | 1.01 (0.85, 1.21) | 1.72 (0.82, 3.60) |
| Quartile 3 | 0.98 (0.82, 1.18) | 1.66 (0.91, 3.02) |
| Quartile 4 | 1.02 (0.86, 1.21) | 1.94 (1.03, 3.66) |
| *P*-trend^c^ | 0.7 | 0.1 |
| IL-18 BP |  |  |
| Quartile 1 | 1.00 (Reference) | 1.00 (Reference) |
| Quartile 2 | 1.02 (0.87, 1.20) | 0.82 (0.58, 1.16) |
| Quartile 3 | 1.14 (0.98, 1.33) | 0.91 (0.65, 1.28) |
| Quartile 4 | 1.13 (0.96, 1.32) | 0.76 (0.55, 1.05) |
| *P*-trend^c^ | 0.09 | 0.1 |
| sICAM1 |  |  |
| Quartile 1 | 1.00 (Reference) | 1.00 (Reference) |
| Quartile 2 | 1.08 (0.93, 1.25) | 0.88 (0.61, 1.28) |
| Quartile 3 | 1.03 (0.88, 1.21) | 0.76 (0.55, 1.05) |
| Quartile 4 | 1.02 (0.87, 1.20) | 0.84 (0.61, 1.15) |
| *P*-trend^c^ | 1.0 | 0.3 |
| Factor D |  |  |
| Quartile 1 | 1.00 (Reference) | 1.00 (Reference) |
| Quartile 2 | 1.08 (0.92, 1.26) | 1.17 (0.52, 2.63) |
| Quartile 3 | 0.99 (0.83, 1.18) | 1.54 (0.75, 3.17) |
| Quartile 4 | 1.09 (0.93, 1.29) | 1.31 (0.63, 2.73) |
| *P*-trend^c^ | 0.5 | 0.4 |
| sEng |  |  |
| Quartile 1 | 1.00 (Reference) | 1.00 (Reference) |
| Quartile 2 | 1.07 (0.91, 1.27) | 1.02 (0.73, 1.43) |
| Quartile 3 | 1.21 (1.04, 1.41) | 0.90 (0.62, 1.31) |
| Quartile 4 | 1.10 (0.94, 1.30) | 1.08 (0.74, 1.57) |
| *P*-trend^c^ | 0.2 | 0.8 |
| CRP |  |  |
| Quartile 1 | 1.00 (Reference) | 1.00 (Reference) |
| Quartile 2 | 0.92 (0.78, 1.07) | 0.88 (0.45, 1.72) |
| Quartile 3 | 0.99 (0.85, 1.16) | 1.15 (0.56, 2.36) |
| Quartile 4 | 0.97 (0.84, 1.13) | 0.97 (0.51, 1.86) |
| *P*-trend^c^ | 0.9 | 1.0 |
| C5a |  |  |
| Quartile 1 | 1.00 (Reference) | 1.00 (Reference) |
| Quartile 2 | 0.96 (0.82, 1.12) | 0.94 (0.65, 1.38) |
| Quartile 3 | 0.97 (0.83, 1.13) | 1.18 (0.82, 1.71) |
| Quartile 4 | 0.95 (0.81, 1.11) | 0.97 (0.68, 1.38) |
| *P*-trend^c^ | 0.6 | 0.8 |

^a^ Estimates were obtained from log-binomial models. Modified Poisson models with robust variance estimation were used to handle model convergence issues whenever necessary. Inadequate and excessive gestational weight gain was defined as < 90% and > 125% percent adequacy, respectively, based on the Institute of Medicine guidelines. The reference outcome for inadequate gestational weight gain included adequate and excessive gestational weight gain; the reference outcome for excessive gestational weight gain included adequate and inadequate gestational weight gain. Ang-1, angiopoietin-1; Ang-2, angiopoietin-2; Angptl3, angiopoietin-like 3; C5a, complement component C5a; CI, confidence interval; CRP, C-reactive protein; Factor D, complement factor D; GWG, gestational weight gain; IL-18 BP, interleukin-18 binding protein; IL-1β, interleukin-1 beta; MCP-1/CCL2, monocyte chemoattractant protein-1; MIPβ/CCL4, macrophage inflammatory protein-1 beta; PGF, placental growth factor; RR, risk ratio; sEng, soluble endoglin; sFlt-1, soluble fms-like tyrosine kinase 1; sICAM1, soluble intercellular adhesion molecule-1; sTNFR2, soluble tumor necrosis factor receptor 2; VEGF-A, vascular endothelial growth factor.

^b^ All models were adjusted for maternal age at enrollment (years), maternal education level (0 to 4 years, 5 to 7 years, 8 to 11 years, and $\geq$ 12 years), marital status (married or not), maternal occupation (employed or not), household wealth index (quintiles), total energy intake (kcal/d), intervention assignment (multiple micronutrient supplementation or control), and first-trimester BMI category (underweight, normal-weight, or overweight/obese). Missing data on maternal occupation and total energy intake were accounted for by using the missing indicator method.

^c^ Computed by assigning the median concentration of each quartile to participants in the corresponding quartile as a continuous variable.
