# Supplementary material for: Plasma concentrations of leptin at mid-pregnancy are associated with gestational weight gain among pregnant women in Tanzania: a prospective cohort study
Source: BMC Pregnancy Childbirth. 2021 Oct 6;21:675. doi: 10.1186/s12884-021-04146-0 (PMC8495974; doi:10.1186/s12884-021-04146-0)
Supplement: Supplementary file 3 — Additional file 3:. [file 12884_2021_4146_MOESM3_ESM.docx]

**Additional file 3** Potential interactions of leptin and chitinase-3-like protein-1 with maternal characteristics on gestational weight gain in a cohort of pregnant women in Dar es Salaam, Tanzania, 2001-2004^a,b^

|  | ***P*-interaction^c^** | |
| --- | --- | --- |
|  | Leptin | CHI3L1 |
| Gestational weight gain percent adequacy |  |  |
| Prenatal regimen^d^ | 0.2 | 1.0 |
| First-trimester BMI category^e^ | 0.02 | < 0.001 |
| Maternal anaemia status at enrollment^f^ | 0.3 | 0.8 |
| Maternal stature^g^ | 0.5 | 0.2 |
| Inadequate GWG |  |  |
| Prenatal regimen | 0.2 | 0.9 |
| First-trimester BMI category | 0.8 | 0.001 |
| Maternal anaemia status at enrollment | 0.04 | 0.08 |
| Maternal stature | 0.2 | 0.1 |
| Excessive GWG |  |  |
| Prenatal regimen | 0.6 | 0.9 |
| First-trimester BMI category | 1.0 | NA^h^ |
| Maternal anaemia status at enrollment | 0.4 | 0.9 |
| Maternal stature | 0.02 | 0.2 |

^a^ Estimates were obtained from linear and log-binomial models for the continuous and binary metrics of gestational weight gain, respectively; modified Poisson models with robust variance estimation were used to handle model convergence issues whenever necessary. Inadequate and excessive gestational weight gain was defined as < 90% and > 125% percent adequacy, respectively, based on the Institute of Medicine guidelines. BMI, body mass index; CHI3L1, chitinase-3-like protein-1; GWG, gestational weight gain.

^b^ All models were adjusted for maternal age at enrollment (years), maternal education level (0 to 4 years, 5 to 7 years, 8 to 11 years, and $\geq$ 12 years), marital status (married or not), maternal occupation (employed or not), household wealth index (quintiles), total energy intake (kcal/d), intervention assignment (multiple micronutrient supplementation or control), and first-trimester body mass index category (underweight, normal-weight, or overweight/obese), and the potential effect modifier examined (if not already included). Missing data on maternal occupation and total energy intake were accounted for by using the missing indicator method.

^c^ Computed by including the main effects and a cross-product term of the protein with the potential effect modifier.

^d^ Multiple micronutrient supplementation or control.

^e^ Underweight, normal-weight, or overweight/obese.

^f^ Any anemia (hemoglobin < 11 g/dL) or no anemia.

^g^ < 150 cm or ≥ 150 cm.

^h^ Could not converge using log-binomial or modified Poisson models.
