# Supplementary material for: Plasma concentrations of leptin at mid-pregnancy are associated with gestational weight gain among pregnant women in Tanzania: a prospective cohort study
Source: BMC Pregnancy Childbirth. 2021 Oct 6;21:675. doi: 10.1186/s12884-021-04146-0 (PMC8495974; doi:10.1186/s12884-021-04146-0)
Supplement: Supplementary file 4 — Additional file 4:. [file 12884_2021_4146_MOESM4_ESM.docx]

**Additional file 4** Associations of leptin and chitinase-3-like protein-1 with gestational weight gain percent adequacy by first-trimester body mass index category in a cohort of pregnant women in Dar es Salaam, Tanzania, 2001-2004^a,b^

|  | ***n*** | **Quartile 1** | **Quartile 2** | **Quartile 3** | **Quartile 4** | ***P*-trend^c^** | ***P*-interaction^d^** |
| --- | --- | --- | --- | --- | --- | --- | --- |
|  |  | Mean difference  (95% CI) | Mean difference  (95% CI) | Mean difference  (95% CI) | Mean difference  (95% CI) |  |  |
| Leptin |  |  |  |  |  |  |  |
| Underweight | 120 | 0.00 (Reference) | 2.92 (-8.40, 14.23) | 13.52 (-0.80, 27.84) | 9.44 (-5.46, 24.34) | 0.10 | 0.02 |
| Normal-weight | 748 | 0.00 (Reference) | 4.65 (-1.43, 10.72) | 3.86 (-2.24, 9.96) | 7.01 (0.68, 13.34) | 0.054 |  |
| Overweight/obese | 133 | 0.00 (Reference) | 1.04 (-56.47, 58.55) | 38.32 (-14.00, 90.64) | 40.78 (-9.00, 90.56) | 0.041 |  |
| CHI3L1 |  |  |  |  |  |  |  |
| Underweight | 120 | 0.00 (Reference) | 8.52 (-4.74, 21.79) | 18.73 (4.21, 33.25) | 14.63 (1.82, 27.45) | 0.039 | < 0.001 |
| Normal-weight | 749 | 0.00 (Reference) | -1.76 (-7.91, 4.39) | 0.05 (-5.97, 6.08) | -0.64 (-6.89, 5.61) | 1.00 |  |
| Overweight/obese | 133 | 0.00 (Reference) | -18.76 (-55.79, 18.27) | -19.40 (-57.59, 18.79) | 38.38 (2.38, 74.39) | 0.0028 |  |

^a^ Estimates were obtained from linear models. CHI3L1, chitinase-3-like protein-1; CI, confidence interval.

^b^ All models were adjusted for maternal age at enrollment (years), maternal education level (0 to 4 years, 5 to 7 years, 8 to 11 years, and $\geq$ 12 years), marital status (married or not), maternal occupation (employed or not), household wealth index (quintiles), total energy intake (kcal/d), and intervention assignment (multiple micronutrient supplementation or control). Missing data on maternal occupation and total energy intake were accounted for by using the missing indicator method.

^c^ Computed by assigning the median concentration of each quartile to participants in the corresponding quartile as a continuous variable.

^d^ Computed by including the main effects and a cross-product term of the protein with the potential effect modifier.
