# Supplementary material for: Plasma concentrations of leptin at mid-pregnancy are associated with gestational weight gain among pregnant women in Tanzania: a prospective cohort study
Source: BMC Pregnancy Childbirth. 2021 Oct 6;21:675. doi: 10.1186/s12884-021-04146-0 (PMC8495974; doi:10.1186/s12884-021-04146-0)
Supplement: Supplementary file 5 — Additional file 5:. [file 12884_2021_4146_MOESM5_ESM.docx]

**Additional file 5** Associations of leptin with inadequate gestational weight gain by maternal anaemia status at enrollment, and associations of chitinase-3-like protein-1 with inadequate gestational weight gain by first-trimester body mass index category, in a cohort of pregnant women in Dar es Salaam, Tanzania, 2001-2004^a^

|  | ***n*** | **Quartile 1** | **Quartile 2** | **Quartile 3** | **Quartile 4** | ***P*-trend^b^** | ***P*-interaction^c^** |
| --- | --- | --- | --- | --- | --- | --- | --- |
|  |  | RR (95% CI) | RR (95% CI) | RR (95% CI) | RR (95% CI) |  |  |
| Leptin^d^ |  |  |  |  |  |  |  |
| Anemic | 679 | 1.00 (Reference) | 0.95 (0.81, 1.11) | 0.87 (0.73, 1.03) | 0.76 (0.62, 0.94) | 0.0084 | 0.04 |
| Not anemic | 263 | 1.00 (Reference) | 1.17 (0.83, 1.66) | 1.14 (0.78, 1.66) | 0.89 (0.59, 1.34) | 0.32 |  |
| CHI3L1^e^ |  |  |  |  |  |  |  |
| Underweight | 120 | 1.00 (Reference) | 0.79 (0.59, 1.05) | 0.55 (0.38, 0.80) | 0.68 (0.52, 0.89) | 0.020 | 0.001 |
| Normal-weight | 749 | 1.00 (Reference) | 1.17 (0.97, 1.40) | 1.09 (0.91, 1.32) | 1.15 (0.95, 1.39) | 0.32 |  |
| Overweight/obese | 133 | 1.00 (Reference) | NA^f^ | NA^f^ | NA^f^ | NA^f^ |  |

^a^ Estimates were obtained from log-binomial models. Modified Poisson models with robust variance estimation were used to handle model convergence issues whenever necessary. Inadequate gestational weight gain was defined as < 90% percent adequacy based on the Institute of Medicine guidelines. CHI3L1, chitinase-3-like protein-1; CI, confidence interval; RR, risk ratio.

^b^ Computed by assigning the median concentration of each quartile to participants in the corresponding quartile as a continuous variable.

^c^ Computed by including the main effects and a cross-product term of the protein with the potential effect modifier.

^d^ Adjusted for maternal age at enrollment (years), maternal education level (0 to 4 years, 5 to 7 years, 8 to 11 years, and $\geq$ 12 years), marital status (married or not), maternal occupation (employed or not), household wealth index (quintiles), total energy intake (kcal/d), intervention assignment (multiple micronutrient supplementation or control), and first-trimester BMI category (underweight, normal-weight, or overweight/obese). Missing data on maternal occupation and total energy intake were accounted for by using the missing indicator method.

^e^ Adjusted for maternal age at enrollment (years), maternal education level (0 to 4 years, 5 to 7 years, 8 to 11 years, and $\geq$ 12 years), marital status (married or not), maternal occupation (employed or not), household wealth index (quintiles), total energy intake (kcal/d), and intervention assignment (multiple micronutrient supplementation or control).

^f^ Could not converge using log-binomial or modified Poisson models.
